# Encountering suicide in primary healthcare rehabilitation: the experiences of physiotherapists

**DOI:** 10.1186/s12888-020-03004-1

**Published:** 2020-12-29

**Authors:** Åse Lundin, Anna Bergenheim

**Affiliations:** 1 Division of Physiotherapy, Department of Health and Rehabilitation, Institute of Neuroscience and Physiology, University of Gothenburg, Gothenburg, Sweden; 2 Närhälsan Rehabilitation Centres, Region Västra Götaland, Gothenburg, Sweden; 3 Research, Development and Education Centre Fyrbodal, Region Västra Götaland, Vänersborg, Sweden

**Keywords:** Physiotherapy, Suicide, Suicidal behaviour, Suicide prevention, Primary healthcare, Rehabilitation

## Abstract

**Background:**

Suicide is a serious public health issue and one of the most common causes of death globally. Suicide has long-lasting impact on personal, relational, community and societal levels. Research has shown that patients often seek help in the primary healthcare system preceding a suicide. Studies exploring the experiences of encountering patients at risk for suicide have been performed among various categories of healthcare personnel, such as nurses and psychiatry residents as well as emergency room staff. There is a lack of research regarding primary healthcare rehabilitation staff, despite the fact that physiotherapists are the third largest health profession in the Western hemisphere and often work with patients experiencing mental health symptoms. The aim of this study was to explore the experiences of encountering patients at risk for suicide among physiotherapists working in a primary healthcare rehabilitation setting.

**Methods:**

Semi-structured interviews were conducted with 13 physiotherapists working in primary healthcare rehabilitation clinics in the Gothenburg area, Sweden. The interviews were recorded on audio and transcribed into written text. A qualitative content analysis was performed on the material collected.

**Results:**

The analysis of the material revealed an overarching theme, *Through barriers and taboos – the physiotherapist finds a way*, with five main categories: *possibilities for identification*, *obstacles in meeting suicide*, *workplace environment matters*, *where does the patient belong*? and *education and experience are keys*.

**Conclusions:**

The present study indicates that physiotherapists in the primary healthcare system encounter patients experiencing suicidality, and they expressed a strong desire to care for both the physical and mental wellbeing of the patients. Despite reporting many barriers, the physiotherapists often found a way to form a meaningful therapeutic alliance with the patient and to ask about possible suicidality in their clinical practice. The result suggests that physiotherapists could play a larger role in working with patients experiencing suicidality in a primary healthcare setting and that they could be viewed as possible gatekeepers in identification as well as referral of these patients into other parts of the healthcare system.

## Background

Suicide is widely recognized as a serious global public health issue with an estimate of 800,000 people dying by suicide annually [1], which makes it one of the most common causes of death. There are also indications that for each adult who dies by suicide there may have been more than 20 others who made an attempt [1]. Both suicides and suicide attempts have long-lasting impacts on personal, relational, community and societal levels [1].

There are multiple factors that elevate the risk of attempting or dying by suicide. There is a significantly increased risk if a patient is disabled, suffers from mental health issues or reports a high occurrence of sick leave from the workplace [2]. Research also shows an elevated risk among individuals with mental or physical disabilities, both on a group level and also related to a specific disorder or diagnosis. This is particularly the case with multiple sclerosis and spinal cord injuries [3]. Concerning mental health, depression is the most common diagnosis in those who die by suicide [4]. An elevated risk has also been detected among patients with traumatic brain injury [5] autism spectrum disorders [6] or chronic pain [7], and among patients afflicted by post-traumatic stress disorder [8].

Previous research has shown that individuals who die by suicide often did turn to the healthcare system for help, with nearly all of the individuals receiving healthcare in the year prior to their death [9, 10]. About 45% of the individuals had been in contact with primary healthcare providers within 1 month preceding their suicide [11]. The World Health Organization [WHO] suggests that focus on suicide prevention should lie with primary healthcare staff, since they are often the entry point to health services and are “available, accessible, knowledgeable, and committed to providing care” [12]*.* This was supported in a recent review that pointed to the primary healthcare system as an important arena for preventing suicidal behaviour, especially in elderly populations [13]. The term “gatekeeper” is often used in suicide research and refers to individuals who regularly interact with potentially suicidal individuals and are available to recognize important behavioural clues that could indicate an elevated risk [14]. In the above-mentioned study by Okolie et al., it was found that community gatekeeper training was identified as an effective intervention in preventing suicidal behaviour in older adults [13].

Studies exploring the experiences of those encountering patients at risk for suicide have been performed among various categories of healthcare personnel, such as nurses, psychiatry residents and emergency room staff [15–17]. Several challenges were identified in these encounters, such as a lack of resources and staff competence as well as low availability of formal or systematic post-suicide mental health programmes for the health workers themselves. There is a lack of research concerning rehabilitation staff and their understanding of patients expressing or experiencing suicidality. One study explored the experiences among counselling support staff in vocational rehabilitation, but it did not include any physiotherapists. The authors found that the staff encountered patients experiencing suicidality but lacked the necessary resources and training to provide appropriate care and support [18]. One recent study was found regarding occupational therapists, a profession closely related to physiotherapy, and their knowledge of suicide-related facts associated with youth suicide. It showed that many occupational therapists lacked the knowledge and comfort to contribute to suicide prevention and reported they had not received proper training or resources to help these patients [19].

Physiotherapy is the third largest health profession in the Western hemisphere and stems from an understanding of health and disease as well as skills in promoting health and treating different kinds of illness, injuries or other health issues [20]. The field of physiotherapy is sometimes overlooked when talking about suicide, since suicidality is often considered a psychiatric specialty. It should be noted that with patients experiencing suicidality there are many ways to ensure survival and that neither physiotherapy nor occupational therapy should be overlooked when a patient is treated for suicidality, for example, in an in-hospital setting [21]. Physiotherapists often possess abilities to work with patients with mental illness such as depression. In assessment and intervention, with the body and movement as central concepts, the physiotherapist can touch upon anatomical, physiological as well as psychiatric and psychosomatic issues [20]. Research has shown that the physiotherapeutic methods such as strength training or aerobic training adjacent to pharmacotherapy are an important part of treating major depressive disorder [22]. Also, yoga and basic body awareness therapy could be considered supplementary treatment options for patients with depressive disorders [23, 24]. Treating the depressive symptoms could be one way for physiotherapists to engage in the care for patients experiencing suicidality, since depression is one of the most common risk factors for suicidal behaviour [4].

In the Gothenburg area in western Sweden it is common to have specialized rehabilitation units adjacent to primary healthcare centres. The primary healthcare rehabilitation clinics employ mostly physiotherapists and occupational therapists, and some units also have access to counsellors, speech therapists or other categories of health personnel. These rehabilitation clinics serve a wide array of patients and are easy to access, due to the requirement to assess the patient within 3 days [25]. Recent research supports the use of physiotherapists as primary assessors of patients with musculoskeletal disorders within the primary healthcare system in Sweden [26]. This raises the question whether primary healthcare physiotherapists could also be viewed as potentially detecting and preventing mental health symptoms such as suicidality.

There is a lack of previous studies concerning the experiences and understandings among physiotherapists in encountering patients who experience suicidality. Physiotherapists are often first-line assessors in primary healthcare and since almost half of the individuals are in contact with primary healthcare providers preceding their suicide [11] it is of large importance to explore physiotherapists’ experiences in meeting these patients. Similar studies regarding closely related healthcare professions have shown a lack of knowledge and training as well as insecurities in meeting patients experiencing suicidality. The present study could increase the knowledge of physiotherapists’ experiences of the matter and provide possibilities to improve the care for patients experiencing suicidality as well as identify resource or training needs for physiotherapists in primary healthcare.

The aim of this research is to explore the experiences of encountering patients at risk for suicide among physiotherapists working in a primary healthcare rehabilitation setting.

## Method

This study explored the *experiences* of clinical physiotherapists, and it was assessed that the favourable method to answer the objective was to use a qualitative method with an inductive approach [27]. The method of analysis was qualitative content analysis according to Graneheim and Lundman. It is a method that describes the manifest content which is close to the text material, but it also comprises the interpretations of the latent material. These interpretations are distant from the text but still near to the participants’ experiences [27].

In qualitative content analysis there is always an interpretation of the material collected, and this interpretation is affected by the researcher’s preunderstanding [28]. The preunderstanding of one of the present researchers is based on 5 years of clinical work as a physiotherapist in the primary healthcare rehabilitation sector, often encountering patients with mental illness or suicidal behaviour. She also has a history of mental illness. The other researcher has conducted several qualitative interview studies and has 18 years of clinical experience in primary healthcare rehabilitation but no previous experience of research concerning patients experiencing suicidality. Both researchers are female.

### Recruitment

The objective of this study was to explore the experiences among physiotherapists working in a primary healthcare rehabilitation setting in the Gothenburg area. One of the largest providers of public primary healthcare in Gothenburg is Närhälsan. Therefore, physiotherapists working at Närhälsan primary healthcare rehabilitation clinics located in Gothenburg and its suburbs were asked to participate. Before the start of the study a signed consent from the participating clinics’ managers was collected. Then, an email asking for participation was sent out along with an information sheet and consent form. A reminder was sent out 3 weeks later and then again 8 weeks after the initial email. A total of 166 emails requesting participation were sent out, and 11 recipients answered the request for participation and went on to be interviewed. One participant working in a public primary healthcare rehabilitation clinic (outside of Närhälsan) found out about the study through a professor at the affiliated university and was included in the study. The participant who was interviewed in a trial interview performed before initiation of the study was also included in the study after consent, making a total of 13 interviews performed and analysed. A convenience sampling method was used, since all physiotherapists who answered the request to participate were selected and went on to be interviewed.

### Data collection

The interviews were carried out during the period September 2018 to March 2019. The interviews took place at the physiotherapists’ workplaces. An audio recorder was used and the interviews were conducted in Swedish. A semi-structured interview guide was developed for this study, see supplementary material. The participants were asked to talk about their experiences related to encountering patients at risk for suicide, through broad questions such as: *Which factors do you believe are of importance for preventing suicide in the primary healthcare sector?* and *How would you describe your current competence to respond to patients who express thoughts about suicide or have attempted suicide?* Follow-up questions were asked to further probe the subject and to be sure that the aim of the study was addressed. The interview guide was tested prior to the study in a trial interview with a physiotherapist at the researcher’s workplace. The physiotherapist who participated in the trial had 5 years of experience working in the primary healthcare system and reported having encountered several patients with suicidal behaviour throughout this period. The interview guide was then slightly modified prior to the start of the study. The trial interview was assessed as good enough to be included in the final analysis and results, bringing to 13 the total number of included interviews. Field notes were not taken during the interviews. The interviews varied in length from 26 to 44 min. For participant characteristics, see Table 1.

**Table 1:** Illustration of the content analysis process, moving from left to right

Meaning unit	Condensed unit	Code	Subcategory	Category
I also have great colleagues to debrief with. I believe we have a very close-knit group and that, that you can grab a hold of and ask pretty much anyone here anything.	We have a close-knit group of colleagues here in which you can debrief and ask anyone anything.	**Support from colleagues**	**The importance of collegiality**	**Workplace environment matters**

Participant checking was conducted continuously during the interview process. The researcher regularly summed up and repeated what the participant had said, to ensure that the given information was perceived properly and allowing the participant to comment. No participant check was performed after the interviews.

### Analysis

The data was analysed using Graneheim and Lundman’s content analysis approach [29]. It is an inductive approach, which means that it is data-driven and not based on an existing theory. Management software was not used in the analysis of the data. The process of analysis began with the transcription of the interviews into written text, which was read several times to obtain a sense of the whole. This text formed the unit of analysis. The text that corresponded to the aim was divided into meaning units that were condensed and labelled with a code, a short phrase capturing the essence of the meaning unit. The various codes were then interpreted and compared in a search for patterns and sorted into 14 subcategories based on their similarities and differences. In that way each subcategory contain codes that share common characteristics. Finally the subcategories were compared with each other and sorted into five higher-level categories. See an illustration of the process in Table 2. The categories and subcategories are an expression of the manifest content of the text, the visible and descriptive that is obvious to the eye [29].

**Table 2:** Participant characteristics (*n* = 13)

		n
Gender	Men	1
	Women	12
Age	20–35	7
	36–55	3
	55 +	3
Work experience in the primary healthcare system	0–3 years	5
	4–15 years	4
	15+ years	4
Highest education	Bachelor’s degree	11
	Master’s degree (1 year)	1
	Master’s degree (2 year)	1

The researchers then took a step back from the material and looked at the entire unit of analysis and what “red thread” ran through the data. In trying to capture the essence of the material and considering the question, “What are these folks trying to tell me?” one overarching theme was formed. The theme can be seen as an expression of the latent content of the text, that is, an underlying meaning emerging through considering what the units, codes and categories are about. Depending on abstraction and the degree of interpretation, it can be captured in one or several themes or subthemes [29].

The analysis was a fluid process throughout, which involved moving back and forth between the whole and parts of the text [29]. The two researchers met regularly during the process of analysis to discuss the material and to go over the emergence of meaning units, codes and categories to ensure a coherent reflection and understanding of the material.

The parts of the analysis regarding transcription, meaning units and codes were all done in Swedish. When the headings of the subcategories, the categories and the overarching theme had been established in Swedish, they were translated to English by the researchers. A colleague fluent in both English and Swedish then translated them back to Swedish again to ensure the accuracy of the translation.

## Results

The recruited participants’ preunderstanding and range of knowledge of the subject of suicide and its prevention was very varied. The majority of the participants had no previous education or training at all or just a short online course or verbal information in suicide prevention. About a third of the physiotherapists had a more extensive knowledge in mental illness as well as suicide prevention, with further education and training in suicide, or a special interest in patients with mental health issues, such as being a part of their unit’s mental health team.

The analysis resulted in 1 overarching theme, 5 categories and 15 subcategories; see Table 3.

**Table 3:** Illustration of the emerged theme, categories and subcategories

Theme Through barriers and taboos – the physiotherapist finds a way	
Category	Subcategory
Possibilities for identification	The physiotherapist as an important gatekeeper
	Time to create alliances
	Mental illness often concealed by physical symptoms
Obstacles in meeting suicide	A lack of knowledge
	A fear of asking and receiving an answer
	Suicide as a taboo
	The body is in focus
Workplace environment matters	The importance of collegiality
	Organizational hindrances
	Negative effects on wellbeing
Where does the patient belong?	A lack of routines for referral
	Difficulties in accessing other health professionals
Education and experience are keys	The value of mentorship
	Work and life experience gives confidence
	Education provides important tools

### Theme

#### Through barriers and taboos – the physiotherapist finds a way

An overarching theme was reached after considering all the material collected and what underlying meaning leapt through it. Throughout the material the physiotherapists described several barriers and taboos that made the clinical encounter of patients experiencing suicidality very difficult. Still, they reported often finding a way to form a meaningful therapeutic alliance with the patient and the courage to ask about their suicidality and acknowledge their struggles. The physiotherapists described how they often went out of their way to make sure the patients at risk made it to the right sector of the healthcare system, even if routines for referral were missing. In general, the physiotherapists expressed being engaged in caring for the patients’ physical and mental wellbeing.

### Possibilities for identification

The physiotherapists reported that they did come across patients with suicidal behaviour and that they were often the first point of entry for the patient into the primary healthcare system. It was not uncommon that the patients initially sought treatment for a physical ailment that later turned out to mask symptoms of mental illness. The physiotherapists had the opportunity to meet with their patients regularly and for longer treatment periods, which allowed them to become close to their patients.

#### The physiotherapist as an important gatekeeper

The physiotherapists reported that in their clinical work they came across patients with suicidal behaviour, and the prevalence of those encounters varied between the physiotherapists, from a few times a year to almost daily. They also often met patients with other mental health symptoms as well as other known present risk factors for suicidal behaviour (such as chronic pain or substance abuse). The physiotherapists reported often being the first point of entry for the patient into the primary healthcare system and that they did recognize themselves as possible gatekeepers to identify patients at risk of suicidal behaviour.

*He showed up and he had been having such a hard time lately. I had never met him before but he was in so much pain and he told me that “I really cannot take this anymore, if I am going to be in this much pain I will … I will throw myself in front of a car.” #5*

#### Time to create alliances

The physiotherapists expressed that they had the possibility to have extended patient visits compared to other categories of health personnel, such as physicians or nurses. They also reported that they often met with patients regularly and for longer treatment periods, sometimes up to several months or a year, which made it possible for the physiotherapists to become close to their patients and create meaningful therapeutic alliances based on trust.

*We are often able to have longer contacts with the patients compared to a physician, for example. And we might have more time to get another … well, when you work out in the training area and you work closely with the patient, that is when you reach a therapeutic relationship in a way and where trust is formed. #4*

#### Mental illness often concealed by physical symptoms

The physiotherapists reported that it was not uncommon during their dialogue and taking of medical history for the patient to express symptoms of mental illness that had not before been mentioned to healthcare personnel. A visit that started out as a sprained ankle could end up being about the patient heading towards depression. The physiotherapists reported that it was important to identify the concealed mental health symptoms, as it was crucial to be able to continue with the rehabilitation process.

*It is not just the ones who seek because of mental illness that experience mental illness. It is brought up with the patients that seek help for issues with their knee joints …*. *It is brought up by almost everyone. #8*

##### Obstacles in meeting suicide

The physiotherapists expressed several obstacles to the identification and support of patients with suicidal behaviour, such as a lack of knowledge as well as a fear of asking about the matter. Suicide and mental illness were described as taboo and discomforting to talk about and as not as tangible or palpable as if the patient suffered from a physical ailment.

#### A lack of knowledge

The physiotherapists expressed difficulties in encountering patients with suicidal behaviour, due to a lack of knowledge of the issue. They regarded their knowledge in meeting this group of patients as low and sometimes even non-existent. They experienced having to engage in dialogue and make mental health assessments that were beyond their capabilities, and that was seen as unfair for both themselves and the patients.

*I don’t believe we have knowledge in that. Just because we have time and are interested, good citizens … well, many times you are told that “That is enough!” just to listen and be present. But that is not what we are educated to do! #11*

#### A fear of asking and receiving an answer

The physiotherapists expressed a great fear of asking their patients about suicidal behaviour. It was rarely asked about directly, and the reported reason for that was the discomfort of receiving an answer that they did not know how to handle. The question was not considered a part of taking the standard medical history, and it was hard to find the right moment to ask as well as to determine the significance of the answer for the current situation or treatment.

*I don’t think it comes naturally to me. The patient usually has to be the one to bring it up. No, I don’t ask about it. I don’t know how many [physiotherapists] do ask? Do you know what I mean? It doesn’t come naturally when taking their medical history. #5*

*Should I really ask that question? How do I handle the answer if I was to receive one? #1*

#### Suicide as a taboo

Several of the physiotherapists mentioned that because suicide has been raised more on a societal level during the last year, its stigma has decreased to some degree. Some of the physiotherapists mentioned the death of artist Tim Bergling (also known as Avicii) by suicide in 2018 as a factor. That suicide is considered taboo showed throughout the interviews, and suicide was described by the physiotherapists as a very charged subject that stirred up a lot of emotions and discomfort in the room. There was a clear comfort threshold to the subject of suicide, and the physiotherapists found it significantly harder to talk about with the patients compared to other mental health symptoms.

*You can also ask if they have experienced other types of mental issues, if they have felt depressed and so on. That is not considered very taboo. But asking if you have had thoughts about ending your own life … well, that is something else. That feels completely different. #4*

#### The body is in focus

The physiotherapists expressed that their focus was usually on physical issues and looking for structural or anatomical anomalies. The focus on the body was viewed as traditionally physiotherapeutic, and the physiotherapists reported that in the clinical work, physical issues were more palpable and did not require as much emotional involvement.

*It is a lot easier if they have a knee … a knee joint that needs replacing. It becomes so much more tangible. #1*

*And then I also believe that the hard part is … as a physiotherapist it might be very clear what to do about a knee or a shoulder, for example. But how do you go about mental illness? #12*

### Workplace environment matters

The workplace and its environment were described as essential by the physiotherapists and could help or hinder their daily clinical life. Having access to colleagues to debrief with was valuable and viewed as a source of support. There were several organizational factors reported that affected the physiotherapists’ daily work negatively as well as having adverse effects on their health and wellbeing.

#### The importance of collegiality

The importance of having access to colleagues as well as good support from the direct manager was clear throughout the interviews. The physiotherapists reported that colleagues were the place to turn to in matters regarding difficult patient cases, as a source of exchange of knowledge and experiences. Colleagues were also used for debriefing and emotional support after meeting a patient who had expressed thoughts of suicide or, as in one participant’s case, where a patient died by suicide. Some of the physiotherapists reported that they did not have access to other physiotherapists in the same clinic and instead sought support from colleagues of other professions or with physiotherapist colleagues in other parts of the healthcare system.

*I also have great colleagues with whom I can debrief. I think we have a very, very close group and that …. It does not have to be someone that works with mental illness but you can grab a hold of anyone. #6*

#### Organizational hindrances

There were several organizational hindrances reported that affected the physiotherapists’ daily clinical work. The biggest hurdle described was a lack of time to sit down with patients expressing mental issues. Although the physiotherapists also reported the possibility of having longer patient visits than were possible for other categories of health staff, there was often not enough time during the visits to go into the subject of mental health, and there was not enough time between patients to debrief and collect further information if needed.

Suicide was generally not talked about at the workplace, and the physiotherapists reported a perceived lack of understanding from those governing the regional healthcare system. They raised significant criticism of the compensation scheme, in which the longer time needed for meeting mental illness was not compensated for. There was also a reported sense among the physiotherapists that mental health, and suicide specifically, is not an area where money and resources are focused within the healthcare system in general.

*We have such high demands on us to perform and accomplish a great deal during our days. You are very focused on managing your day, really. That means you might not be able to settle down or reflect that much afterwards. So, the time pressure I think is … or the pressure in general I think is a factor that makes it difficult. #3*

#### Negative effects on wellbeing

The physiotherapists expressed that encountering patients with suicidal behaviour could sometimes be distressing and take a toll on the physiotherapists’ own health. They described leaving these visits feeling exhausted, and they sometimes experienced physical symptoms such as muscle tension or shakiness and at times also took these feelings home at the end of the day. The physiotherapists who had patients who died by suicide described an immensely hard situation to deal with.

*After having one or more of those patients that day, I feel that it does something to me. I get very depressed myself. It’s hard. #8*

*I do not know if that participant had died by suicide. But I suspect that was the case. So that was something that was very hard. Could anything more have been done? #4*

### Where does the patient belong?

The physiotherapists reported experiencing long waiting times and inaccessibility for the patient to meet with other healthcare professionals. It was also difficult for the physiotherapists to guide a patient at risk for suicide to the right sector of the healthcare system, due to a lack of referral routines.

#### A lack of routines for referral

The physiotherapists reported a lack of and a request for routines for referral of the patients beyond the primary healthcare sector. They did not know how to guide a patient at risk for suicide further through the healthcare system, and the physiotherapists felt an uneasiness in leaving a patient who had expressed symptoms of a severe mental illness, without making sure they were taken care of. There was also a great worry for the physiotherapists whether they had acted the right way or not in trying to refer a patient further.

*I see a problem in that I don’t really know where to turn. What if it is really urgent – where do I turn? That would be interesting to talk or learn more about. #10*

*Of course, you find yourself thinking, “Did I do the right thing?” Will something happen now or not? Will the person in question make that call and ask for help? #7*

#### Difficulties in accessing other health professionals

The physiotherapists expressed several difficulties regarding not only health personnel but also patients’ ability to access other parts of the healthcare system. They reported extensive wait times for the patients to see a specialist as well as a lack of psychologists in primary healthcare, meaning the patients were put on endless waiting lists. It was described as comforting if the patient already had initiated contact with a specialist physician or psychologist. But several physiotherapists described attempts to facilitate contact with physicians, in which they had been rejected or never called back. On one occasion a patient who – at the physiotherapist’s request – sought care in the emergency psychiatric unit was rejected there, causing great distress to both the patient and the physiotherapist.

*It is not always easy to get hold of a psychologist … or whoever the patient may need. Instead, the patient will have to wait for three months before seeing someone, and then it might already be too late. #1*

### Education and experience are keys

Work experience as well as becoming older was mentioned by the physiotherapists as important factors in creating confidence to encounter patients experiencing suicidality. Collegial mentorship and education in suicide prevention were reported as highly valued, and there was a desire to obtain this among the physiotherapists who did not have access to it.

#### The value of mentorship

Mentorship and clinical guidance from both other professions as well as more senior physiotherapists was reported as highly valued. Those who had access to mentorship expressed a wish to extend the sessions and have them more often, and the ones who reported not having access to mentorship had a wish to obtain it. Mentorship and clinical guidance were reported as central when starting work as a recently graduated physiotherapist. A wish to consult with colleagues more often to get insight into how they worked with this group of patients was also expressed by the physiotherapists.

*Nursing students receive mentorship in their profession during several semesters along with clinical training …. You get tutoring in what you experience, what you observe. As physiotherapists we don’t have that, at all. It is crap! Not even a single minute! #9*

#### Work and life experience give confidence

Experience was found to be an important aspect in how confident the physiotherapists felt in encountering patients with suicidal behaviour. Work experience and having met many patients with mental illness were reported to facilitate and made it easier to engage such a topic during the visit. Life experience in general and becoming older were mentioned as confidence factors in dealing with suicide, as well as having their own previous experience of mental illness.

*With the experience of the years you have worked, but also with your own age, you become better at handling these things in a different way. #7*

#### Education provides tools

Mental illness in general was reported by the physiotherapists as usually covered in the university curricula offered in physiotherapy programmes. But information regarding suicide and its prevention specifically was reported as being often left out, or the material given was outdated. The physiotherapists expressed a concern that if they were expected to encounter this group of patients, they also needed the right education to do so. The physiotherapists who had received education in suicide prevention felt that it gave them an increased awareness as well as practical tools for encountering patients with suicidal behaviour. The physiotherapists who had not had further education expressed a wish to obtain this, if given the opportunity.

*You gained more tools to ask about it in a different way. It somehow gave me the courage to ask how far in their thoughts they had come … if they only thought that it might happen at some point in the future, or if they had actually decided how to do it. How far they had climbed the [suicidal] staircase. And I hadn’t really thought about it that way before. #7*

## Discussion

The aim of this study was to explore the experiences of encountering patients at risk for suicide among physiotherapists working in a primary healthcare rehabilitation setting. In general, there is a lack of past or current research regarding physiotherapists and their encounters with and experiences of suicide, especially in physiotherapists working in the primary healthcare sector. However, the results of the present study are to a large extent coherent with other health professionals’ experiences in previous research and literature in regard to challenges, possibilities, organizational structures and social constructions of suicide.

After all the collected data were analysed, one theme emerged: *Through barriers and taboos – the physiotherapist finds a way*. The theme developed successively through the interviews as well as during the data analysis, where the researchers noticed similar stories from several of the physiotherapists. These stories mainly involved the hardships and obstacles that surrounded the clinical encounter of patients experiencing suicidality. Most of these hardships were related to organizational or educational matters and the fact that suicide was considered a taboo subject to bring up on both a clinical and personal level. During the interviews the researchers saw yet another pattern in the narratives: the physiotherapists somehow found ways through the reported difficulties and were able to acknowledge their patients despite these struggles. The engagement that the physiotherapists expressed with respect to the patients was vast, and they often went out of their way and put extra time and effort into making sure the patient would be all right, thereby curbing and climbing many of the obstacles they had previously described. This large engagement with patients experiencing suicidality has been reported in other sectors within the healthcare system, such as by nurses working in the emergency room, where the research data is permeated by a great compassion and a need to help [17].

The category *Possibilities for identification* included several aspects that appeared to play a role in the physiotherapists’ abilities to detect patients at risk for suicide in the clinical practice. One important aspect is that the physiotherapists could be viewed as possible gatekeepers, referring to individuals who regularly interact with potentially suicidal individuals and are available to recognize important behavioural clues [14]. The physiotherapists in this study fitted the gatekeeping role well, they were often the first contact in the primary healthcare system, they had more time allowed for the visits than the physicians or nurses and they often picked up on the mental issues that were concealed behind the physical issues for which the patient initially sought treatment. This finding is supported by the WHO, which claims that primary healthcare staff should be viewed as important elements in suicide prevention, since they often have long and close contact with the community. They also serve as a link between the community and the healthcare system as well as often being an entry point to the health services for those in distress [12].

The present study suggests that not only physicians, nurses and psychologists but also physiotherapists could be viewed as potentially identifying individuals at risk for suicide. Time was also an important factor in encountering these patients and the possibility of identifying and detecting suicidal behaviour. Several of the physiotherapists reported that although time was never enough, they often had *more* time than other health professions, which made it possible for them to become close and create meaningful therapeutic alliances with their patients.

The importance of creating therapeutic alliances with patients is seen in similar current research as a factor in providing quality care for the patient [30]. Building a strong therapeutic alliance with patients at risk for suicide is vital, since a strong alliance is associated with fewer suicidal thoughts [31]. As a physiotherapist, to fully interact with a patient by bodily resonance and by being present might change the outcome of a patient encounter [32]. Looking at the present study, the understanding and awareness of these concepts might aid the physiotherapists in encountering and detecting a patient with suicidal behaviour.

The physiotherapists in the present study reported several barriers that hindered them in their encounter of patients expressing suicidality, which resulted in the category *Obstacles in meeting suicide.* That suicide is considered a taboo subject became evident throughout the interviews. It is a subject that raises discomfort in many individuals and societies, and taking one’s own life was a punishable crime in Sweden until 1864 [33]. Today it is talked about more and more on a societal as well as a healthcare level, and the physiotherapists spoke freely about the matter when interviewed. Despite this, they did report it to be a large challenge to talk about suicide with patients in an environment where the physiotherapists didn’t know how the subject would be received. There was fear and anxiety of asking about the patients’ suicidal ideations, mostly due to the risk of receiving an answer that the physiotherapists did not know how to handle. This result is seen in other recent research which reported that the difficulties may actually originate not in asking the question but rather in the therapist’s fear of receiving a positive answer (the patient reporting suicidal ideation) [34]. There is also a risk that healthcare personnel’s anxieties and fears curb the identification and treatment of suicidal ideation in patients whom they encounter, since fear drives avoidant behaviour. “If you do not want to know if your patient has a fever, do not take her temperature”, as the old medicine adage says [35].

A lack of knowledge about suicide was reported by the physiotherapists in the present study, and that they had experienced situations in which they were to make assessments about suicidality beyond their capabilities and competence. The same challenges regarding a lack of knowledge are also described by the participants in research regarding personnel working in a vocational rehabilitation setting in their encounters with patients experiencing suicidality [18]. Research concerning nurses working in the emergency department reported a very similar challenge, where the nurses experienced that the lack of skills left them with a feeling that they were out of their depth [17].

A downside to the physiotherapists’ engagement with patients experiencing suicide and their attempts to find solutions was seen in the category *Workplace environment matters.* The physiotherapists reported bringing their work stress home with them at the end of the day or having to squeeze these patients in to a schedule where there really was no time. This paved the road for negative health effects for the physiotherapists themselves. One physiotherapist expressed thoughts about leaving the primary healthcare sector and looking for a job elsewhere, since the work situation had so many negative effects on their health and life outside of work. One reason for potentially leaving the position was a reported lack of understanding within the organization. The regionally governed healthcare system had set the bar at a level where the experienced daily work stress was close to unbearable. The physiotherapists were also frustrated with a system they viewed as promoting short patient visits, thus limiting optimal and patient-centred work. With the majority of the physiotherapists reporting this level of stress, there was a clear frustration in the clinical setting, which is coherent with other primary healthcare research, where, for example, nurses are reported to skip lunches due to overbooked schedules [30].

One participant who had experienced a patient’s probable death by suicide reported an onset of enormous stress from the situation, including feelings of guilt as well as of not having enough time or resources to meet or support this patient’s needs. That organizational factors affect the health and wellbeing of healthcare personnel who experience inpatient suicide is seen in previous research within several sectors of healthcare. In psychiatric residents the formal institutional support for the trainees who experienced a patient’s death by suicide was described as having major shortcomings and could lead to post-traumatic symptoms [15]. In nursing research, inpatient suicide has been correlated with significant mental distress for the caregiver, and there is a reportedly low availability of systematic post-suicide mental healthcare programmes for healthcare personnel [16]. Instead, one of the largest sources of support after experiencing a patient’s death by suicide was collegial peer support [15]. As reported in the present study, the majority of the physiotherapists praised their co-workers and found huge relief in having a close relationship with them. The physiotherapists in the present study also stated that they would have found it hard to cope with their work situations without having access to this kind of debriefing after tough patient encounters. Using colleagues as one of the most important factors in the work environment corresponds to previous research showing that sharing mutual trust and respect with colleagues helped greatly to create job satisfaction as well as learning opportunities [36]. However, using colleagues as one of the most important factors in the work environment should not be considered enough: it is essential that healthcare services invest in systematic post-suicide mental healthcare programmes for healthcare personnel.

Previous research has highlighted how the referral of a patient at risk for suicide from the vocational rehabilitation sector can be an obstacle when the community lacks resources for where and how to refer the patient [18]. These findings are coherent with the present study, which resulted in the category *Where does the patient belong?* The participating physiotherapists expressed significant challenges in guiding a patient at risk for suicide through the healthcare system as well as how to get hold of other health professionals. Patients were also reported to have been rejected at the emergency department, causing significant distress to both patient and physiotherapist. The separation between outpatient and inpatient care causes a communication barrier that in similar studies has been shown to result in misjudgement about patients’ suicidality [35].

Work experience as well as becoming older were mentioned by the physiotherapists as important factors in creating confidence to encounter patients experiencing suicidality, and this resulted in the category *Education and experience are keys.* Among the physiotherapists who had not received any education in suicide, there was a noticeable desire and need for education, preferably in the bachelor level physiotherapy programme but also as further training in the clinic. This is in line with other research on health personnel in vocational rehabilitation where there was a lack of training and education and where the participating staff expressed a desire for training in the field of suicide, without which they would not be able to provide proper care for their patients [18]. Education seems to pay off in the clinical setting. Research regarding gatekeeping educational programmes for nurses has shown an increased awareness of suicide warning signs among patients [14]. The majority of the physiotherapists in the present study expressed a substantial lack of knowledge in encountering patients expressing suicidality as well as a concern that if they are expected to encounter this group of patients, they also need the right education to do so. The focus going forward needs to be in the education of physiotherapists to know how and what to do when encountering these patients.

### Strengths and limitations

Qualitative content analysis was chosen as the method for analysis and interpretation of the material collected. Selecting the most suitable meaning units was a challenging part in achieving credibility. Too broad a unit might capture several different meanings and too narrow a unit could result in fragmentation [29]. The two researchers spent time discussing the material and result back and forth, and adjustments were made to the categories and subcategories throughout the entire process.

One challenge regarding the study’s dependability was that the researcher who performed the data collection had not used a qualitative research method before. Being new to the method meant there was probably a longer process of evolving throughout the interviews than expected for researchers who are more familiar with the technique. It was also very important to remain aware of the researchers’ preunderstanding throughout the process [27]. The researcher who performed the recruitment, the data collection and the major part of the analysis has a large interest in suicide prevention on both a professional and personal level and had to constantly question the interpretation of data, results and conclusions. The other researcher played a large role in not having that same prior knowledge or background.

In this research the researcher who conducted the interviews was close to both the field and the material and also took the position of an insider by having the same educational background and working in the same region and sector of healthcare as the participants. Studying the familiar made for an easier entry into the research topic, especially when it came to recruiting the participants, and it also affected the participants’ way of talking to the researcher, who thus was able to understand underlying meanings or situations, even if the participants had only hinted at it.

Another limitation of the present study was that no research journal was kept or field notes taken during the preparation, interviewing or analysis. This is a measure recommended to maintain reflexivity, since keeping a journal makes “self-supervision” possible, and a trail of the researcher’s emotional reactions and reasoning can be created [37]. If the researchers were to replicate or perform a similar study, one early act would be that of creating a research journal as well as taking field notes during the interviews.

Regarding methods of confirming the data, no methods were used to collect data other than the semi-structured interviews between the researcher and the participants. Adding another method of data collection such as observational data or focus group interviews with the participants was not done in this study, but might have resulted in a greater variance and a different understanding of the objective.

The transferability of the present study depends on the entire process from participant recruitment through to collection and analysis of the data and the context and culture in which the research was conducted [29]. These variables have been described thoroughly by the researchers, which creates a basis for other researchers who are interested in looking into the field further or reproducing the study in other settings.

The group of participants had a diversity of experience and age, which was considered a strength of the study. The majority of participants were women, and one explanation is that physiotherapy is predominantly a female work position in Sweden. In 2014, 79% of the active physiotherapists in Sweden were women [38], a proportion that corresponds to the participants in the present study. During the recruitment, two reminders were sent out, after which no more informants responded. A convenience sampling method was used, and all of the interested physiotherapists were included in the study and went on to be interviewed. In qualitative content analysis research there is always a concern regarding the number of participants and how it is not possible to suggest a specific number of interviews to be performed [27]. The final number of participants included in this study was 13, and although the richness of the collected data was considered to be sufficient to answer the study purpose, the inclusion of more participants in the study might have added new perspectives on the subject.

The present study was performed in the Swedish primary healthcare sector in Gothenburg. The compensation system was reported as a large factor behind the perceived stress and time restraints. The current system is based on the patient’s ability to choose their preferred primary health caregiver. This system has received criticism from other health professionals within the primary healthcare, such as nurses, as focus tends to fall on economic benefit instead of competence and skill [39]. The three largest regions in Sweden, Stockholm, Västra Götaland and Skåne, all use this or a similar compensation system [40], but inclusion of some of the smaller regions that use other methods would have added transferability to the results of this study.

All of the participants worked in the greater Gothenburg area, and several of them worked in larger clinics with around 10 to 15 other physiotherapists as colleagues. Again, not including smaller cities or rural areas outside of Gothenburg is seen as a limitation of this study. It is not uncommon in more rural areas of Sweden that physiotherapists in primary healthcare work alone without access to colleagues within the same field, or that they have other factors regarding resources or referral systems that would have changed the results and outcome of this study.

The researcher who performed the interviews was familiar with three of the participants briefly through previous study or work, which might have affected the participants’ motivation to open up during the interviews. However, the participants knew who was performing the interviews when they signed up to the study.

### Implications for future research

There are many suggestions for future research and areas of development within the interdisciplinary field of physiotherapy and suicidology. It is important to understand the patient perspective as well: what are the experiences of patients at risk for suicide who have met and been assessed by a physiotherapist in a gatekeeper position? Looking at a societal level, could a different physiotherapy role alleviate the work or support other groups of health staff in the primary healthcare system in identifying patients at risk for suicide? Previous research has shown that education in suicide prevention often raises health staff’s confidence. How would an educational campaign directed towards primary healthcare physiotherapists affect their self-perceived competence levels?

## Conclusions

It is generally acknowledged that the practice of suicide prevention is a task for everyone [21]. Since mental health in general and suicide specifically are more talked about on a societal level today, there might be a growing place for physiotherapists to take part in that development in awareness, detection and treatment. However, the role and responsibilities of physiotherapists when it comes to meeting patients experiencing suicidality remains largely an unexplored area. The results of the present study indicate that physiotherapists in the primary healthcare system encountered patients with suicidal behaviour and could be viewed as possible gatekeepers in identifying individuals at risk, a role that is more often assigned to other healthcare professions.

The physiotherapists fitted the gatekeeping role well; they often found a way to make a meaningful alliance with the patients and asked about their possible suicidality, despite taboos, educational issues, potential health hazards and the organizational obstacles reported to hamper them in the clinical setting. The physiotherapists expressed a strong desire to care for the patient’s entire wellbeing, strengthening the role of the physiotherapists as health professionals who are able to work with both physical and mental issues in patients.

The present study’s results of possibilities and obstacles faced in primary healthcare regarding encountering patients experiencing suicidality are both coherent with and confirm earlier research regarding other health professions. It is vital to address these findings if physiotherapists are to play a larger role in working with patients experiencing suicidality in the primary healthcare sector. This is mainly to provide better quality of care for the primary healthcare patients and to help curb society’s high suicide numbers, but also important to address to increase competence as well as ensure health and wellbeing within the physiotherapy workforce.

## Supplementary Information

**Additional file 1.**

## Data Availability

All data collected or analysed during the current study are also available from the corresponding author on reasonable request.

## Abbreviation

WHO: World Health Organization

## References

[1] WHO (2019). Suicide prevention.

[2] Qin P, Agerbo E, Mortensen PB (2003). Suicide risk in relation to socioeconomic, demographic, psychiatric, and familial factors: a National Register–Based Study of all suicides in Denmark, 1981–1997. Am J Psychiatr.

[3] Giannini MJ, Bergmark B, Kreshover S, Elias E, Plummer C, O'Keefe E (2010). Understanding suicide and disability through three major disabling conditions: intellectual disability, spinal cord injury, and multiple sclerosis. Disability Health J.

[4] Henriksson MM, Aro HM, Marttunen MJ, Heikkinen ME, Isometsa ET, Kuoppasalmi KI (1993). Mental disorders and comorbidity in suicide. Am J Psychiatry.

[5] Teasdale TW, Engberg AW (2001). Suicide after traumatic brain injury: a population study. J Neurol Neurosurg Psychiatry.

[6] Segers M, Rawana J (2014). What do we know about suicidality in autism spectrum disorders? A systematic review. Autism Res.

[7] Racine M. Chronic pain and suicide risk: a comprehensive review. Prog Neuropsychopharmacol Biol Psychiatry. 2018;87(Pt B):269–80. 10.1016/j.pnpbp.2017.08.020. Epub 2017 Aug 26. PMID: 28847525. 10.1016/j.pnpbp.2017.08.020 28847525

[8] Lutwak N, Dill C. PTSD and Risk of Suicide. Mil Med. 2017;182(9–10):1684. 10.7205/MILMED-D-17-00104. PMID: 28885928. 10.7205/MILMED-D-17-00104 28885928

[9] Ahmedani BK, Simon GE, Stewart C, Beck A, Waitzfelder BE, Rossom R (2014). Health care contacts in the year before suicide death. J Gen Intern Med.

[10] De Leo D, Draper BM, Snowdon J, Kolves K (2013). Contacts with health professionals before suicide: missed opportunities for prevention?. Compr Psychiatry.

[11] Luoma JB, Martin CE, Pearson JL (2002). Contact with mental health and primary care providers before suicide: a review of the evidence. Am J Psychiatry.

[12] WHO (2000). Preventing suicide - A resource for primary health care workers.

[13] Okolie C, Dennis M, Simon Thomas E, John A (2017). A systematic review of interventions to prevent suicidal behaviors and reduce suicidal ideation in older people. Int Psychogeriatr.

[14] Tsai W-P, Lin L-Y, Chang H-C, Yu L-S, Chou M-C (2011). The effects of the gatekeeper suicide-awareness program for nursing personnel. Perspectives Psychiatric Care.

[15] Leaune E, Ravella N, Vieux M, Poulet E, Chauliac N, Terra JL (2019). Encountering patient suicide during psychiatric training: an integrative, systematic review. Harvard Review Psychiatry.

[16] Takahashi C, Chida F, Nakamura H, Akasaka H, Yagi J, Koeda A (2011). The impact of inpatient suicide on psychiatric nurses and their need for support. BMC Psychiatry.

[17] Doyle L, Keogh B, Morrissey J (2007). Caring for patients with suicidal behaviour: an exploratory study. Brit J Nurs (Mark Allen Publishing).

[18] Lund EM, Schultz JC, Thomas KB, Nadorff MR, Sias CM, Chowdhury D, et al. "I Honestly Would Not Have Known What to Do": An Exploratory Study of Perspectives on Client Suicide Among Vocational Rehabilitation Support Staff. Omega (Westport). 2017:30222817739215. 10.1177/0030222817739215 29137531

[19] Kirby AV, Terrill AL, Schwartz A, Henderson J, Whitaker BN, Kramer J (2020). Occupational therapy Practitioners' knowledge, comfort, and competence regarding youth suicide. OTJR Occupation Participation Health.

[20] Broberg C, Tyni-Lenné R. Sjukgymnastik som vetenskap och profession.. Legitimerade Sjukgymnasters Riksförbund, wwwfysioterapeuternase. 2009.

[21] Beskow J. Självmord och självmordsprevention. 1 ed. Lund: Studentlitteratur; 2000.

[22] Moraes HS, Silveira HS, Oliveira NA, Matta Mello Portugal E, Araújo NB, Vasques PE, et al. Is Strength Training as Effective as Aerobic Training for Depression in Older Adults? A Randomized Controlled Trial. Neuropsychobiology. 2020;79(2):141–9. 10.1159/000503750. Epub 2019 Oct 28. PMID: 31658460. 10.1159/000503750 31658460

[23] Cramer H, Lauche R, Langhorst J, Dobos G (2013). Yoga for depression: a systematic review and meta-analysis. Depression Anxiety.

[24] Danielsson L, Rosberg S (2015). Opening toward life: experiences of basic body awareness therapy in persons with major depression. Int J Qual Stud Health Well Being.

[25] Krav och kvalitetsboken Vårdval Rehab 2020: Västra Götalandsregionen; [2020-08-10]. Available from: https://www.vgregion.se/halsa-och-vard/vardgivarwebben/uppdrag-och-avtal/vardval-rehab/krav-och-kvalitetsbok/.

[26] Bornhöft L, Larsson MEH, Thorn J (2015). Physiotherapy in primary care triage – the effects on utilization of medical services at primary health care clinics by patients and sub-groups of patients with musculoskeletal disorders: a case-control study. Physiotherapy Theory Practice.

[27] Graneheim U, Lindgren BM, Lundman B (2017). Methodological challenges in qualitative content analysis: a discussion paper. Nurse Educ Today.

[28] Malterud K (2014). Kvalitativa metoder i medicinsk forskning: en introduktion.

[29] Graneheim U, Lundman B (2004). Qualitative content analysis in nursing research: concepts, procedures and measures to achieve trustworthiness. Nurse Educ Today.

[30] Halcomb E, Ashley C (2017). Australian primary health care nurses most and least satisfying aspects of work. J Clin Nurs.

[31] Dunster-Page C, Haddock G, Wainwright L, Berry K (2017). The relationship between therapeutic alliance and patient's suicidal thoughts, self-harming behaviours and suicide attempts: a systematic review. J Affect Disord.

[32] Engelsrud G, Oien I, Nordtug B (2019). Being present with the patient-a critical investigation of bodily sensitivity and presence in the field of physiotherapy. Physiother Theory Pract.

[33] Prisma. Prismas uppslagsbok: Prisma; 1986.

[34] Quinnett P (2019). The role of clinician fear in interviewing suicidal patients. Crisis..

[35] Hendin H, Haas AP, Maltsberger JT, Koestner B, Szanto K (2006). Problems in psychotherapy with suicidal patients. Am J Psychiatry.

[36] Rabie T, Klopper HC, Coetzee SK (2017). Creating positive practice environments in a primary health care setting. Int J Nurs Pract.

[37] Berger R (2015). Now I see it, now I don’t: researcher’s position and reflexivity in qualitative research. Qual Res.

[38] Socialstyrelsen (2016). Allt fler utbildar sig till fysioterapeuter.

[39] Hollman D, Lennartsson S, Rosengren K (2014). District nurses' experiences with the free-choice system in Swedish primary care. Brit J Community Nurs.

[40] Socialstyrelsen. Primärvårdens uppdrag - En kartläggning av hur landstingens uppdrag till primärvården är formulerade. 2016;2016-3-2.

