# Supplementary material for: Encountering suicide in primary healthcare rehabilitation: the experiences of physiotherapists
Source: BMC Psychiatry. 2020 Dec 29;20:597. doi: 10.1186/s12888-020-03004-1 (PMC7771061; doi:10.1186/s12888-020-03004-1)
Supplement: Supplementary file 1 — Additional file 1. [file 12888_2020_3004_MOESM1_ESM.docx]

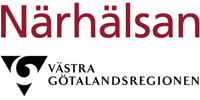


**Experiences**

- While working in primary healthcare rehabilitation, have you encountered patients who have expressed thoughts about suicide or attempted suicide? Could you elaborate on some of these encounters?
- In your experience, how common is it that you encounter patients expressing suicidality in the primary healthcare rehabilitation sector?
- How do you talk about patients experiencing suicidality at your workplace?
- Do you believe there is a fear of asking or talking about suicidality at your workplace?
  - Amongst physiotherapists in general? Amongst other health professions?
- In your experience, is there a difference in how physiotherapists talk about or talk to patients who express suicidality compared to other mental illnesses? In what way?
- Depression is one of the most common risk factors for suicidal behavior, how do you think physiotherapists can aid in the identification or treatment of depression?
- In your opinion, is it important to bring up the subject of suicide within the primary healthcare sector? Would you elaborate on why or why not?
- Which factors do you believe are of importance for preventing suicide in the primary healthcare sector?

**Perceived competence**

- Do you have any formal education or training in suicide prevention and could you elaborate on what it consisted of or what you wish to receive regarding education or training?
- Have you in any other way acquired knowledge about the subject or this group of patients, and if so in what way?
- How would you describe your current competence to respond to patients who express thoughts about suicide or have attempted suicide?
- How do you feel about asking patients about their mental health in general?
  - About suicidality specifically?
- How does talking about suicidality with patients affect you emotionally? How do you debrief after such an encounter?

**Final considerations**

- Do you have any other thoughts or reflections regarding the subject that we have not touched upon and that you would like to highlight?
- Do you have any questions for me before we conclude?
- May I contact you again if something is uncertain during the transcription and analysis of this interview?
